# What's new in heart failure? September 2025

**DOI:** 10.1002/ejhf.70054

**Published:** 2025-10-07

**Authors:** Julian Hoevelmann, Philipp Markwirth, Mert Tokcan, Bernhard Haring

**Affiliations:** ^1^ Department of Internal Medicine III ‐ Cardiology Angiology and Intensive Care Medicine, Saarland University Hospital, Saarland University Homburg Germany; ^2^ HOMICAREM (HOMburg Institute of CArdioREnalMetabolic Medicine), Medical Faculty Saarland University Homburg Germany; ^3^ Cape Heart Institute, Faculty of Health Sciences University of Cape Town Cape Town South Africa; ^4^ Department of Epidemiology and Population Health Albert Einstein College of Medicine Bronx NY USA; ^5^ Department of Medicine IV Clinic Hietzing, Vienna Healthcare Group Vienna Austria

In this column, we want to provide clinicians and researchers with short and concise summaries of recently published articles in the *European Journal of Heart Failure* that we think may be of particular relevance to heart failure (HF) specialists (*Figure* *1*). Key topics of this issue include novel insights into arrhythmia‐induced cardiomyopathy (AIC), profiling of hypotension in HF, the association between clonal haematopoiesis and incident HF, as well as the phenotype‐specific relationship between blood pressure (BP) and cardiovascular outcomes in different HF subtypes.

**Figure 1:**
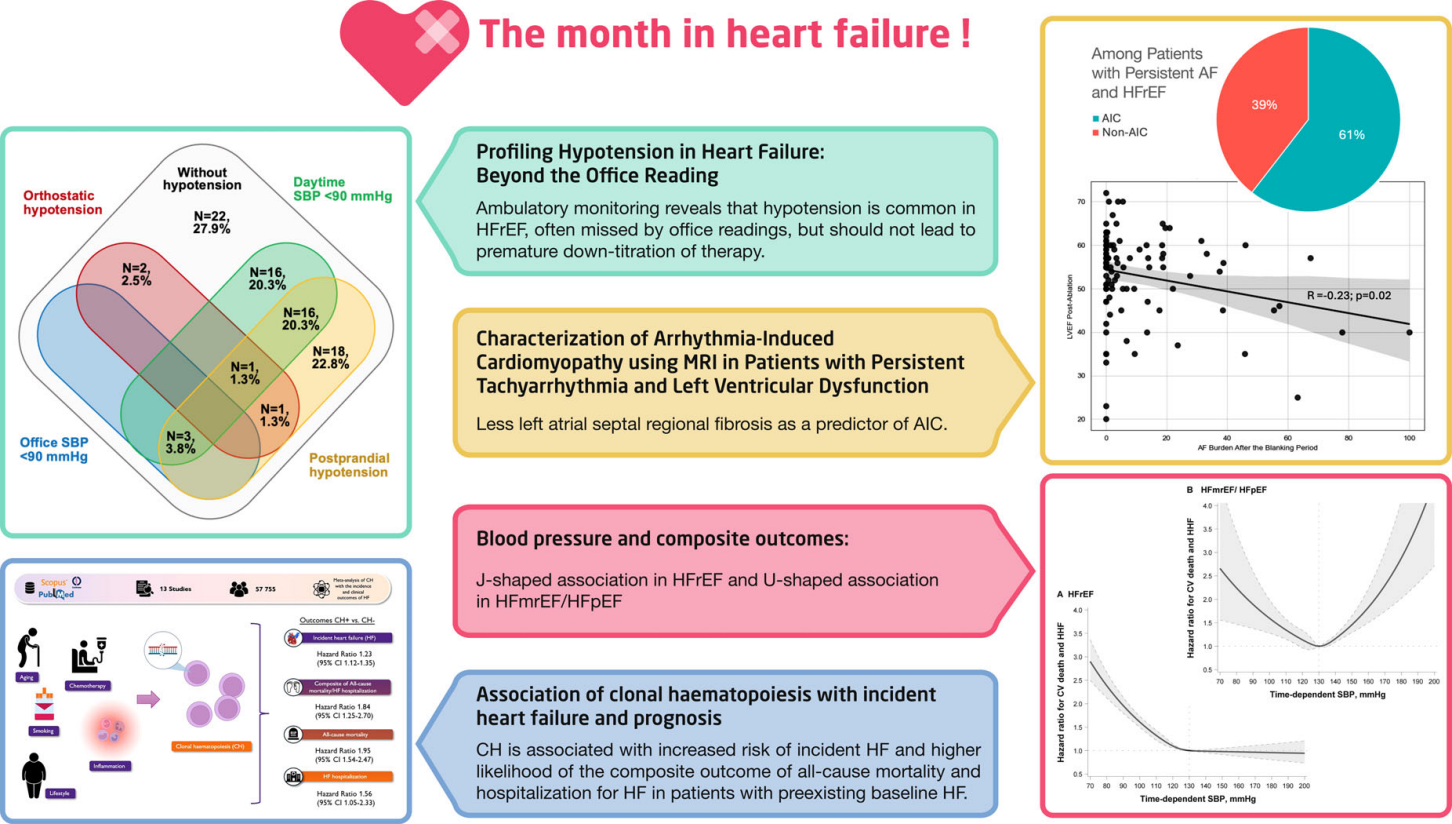
The month in heart failure. AF, atrial fibrillation; AIC, arrhythmia‐induced cardiomyopathy; CH, clonal haematopoiesis; CI, confidence interval; CV, cardiovascular; HF, heart failure; HFmrEF, heart failure with mildly reduced ejection fraction; HFpEF, heart failure with preserved ejection fraction; HFrEF, Heart failure with reduced ejection fraction; MRI, magnetic resonance imaging; SBP, systolic blood pressure.

## Left atrial septal fibrosis as a predictor of arrhythmia‐induced cardiomyopathy

Arrhythmia‐induced cardiomyopathy is a reversible cause of left ventricular systolic dysfunction (LVSD) associated with atrial fibrillation (AF).^1^ In current practice, AIC remains poorly defined and insufficiently characterized, and it is most often identified retrospectively, once left ventricular function improves following adequate rhythm control with antiarrhythmic drugs or AF ablation. Catheter ablation has been shown to improve outcomes in patients with AF and HF.^2^, ^3^, ^4^ However, distinguishing AIC from other primary cardiomyopathies causing LVSD remains a major challenge, as its diagnosis typically depends on retrospective confirmation following recovery of left ventricular ejection fraction (LVEF). In a post‐hoc analysis of the DECAAF II trial, Assaf *et al*.^5^ aimed to evaluate the prevalence and predictors of AIC in patients with persistent AF and LVSD utilizing late gadolinium enhancement cardiac magnetic resonance (LGE‐CMR) imaging.

Among 815 patients undergoing ablation, 119 with LVSD were analysed. At baseline the cohort had a mean LVEF of 39%. Close to two thirds of patients (60.5%) fulfilled criteria for AIC, defined as LVEF recovery to ≥50% with ≥10% absolute improvement or an absolute improvement (≥15%) after ablation. Patients with AIC showed significantly greater LVEF improvement of 19.9 ± 7.6% compared to 4.8 ± 7.5% in non‐AIC patients (*p* < 0.001). Lower AF burden at 12 months post‐ablation correlated with higher LVEF at 3 months post‐ablation (r = −0.23, *p* = 0.02). Using the Youden index, an AF burden of <3.8% was identified as the optimal predictor of AIC status (area under the curve 0.706, *p* = 0.024). LGE‐CMR revealed that AIC patients had significantly less atrial septal fibrosis (12.2% vs. 20.7%, *p* < 0.001), while global left atrial fibrosis burden was not predictive.

In conclusion, the findings of the study provide evidence that in the majority patients with persistent AF the tachyarrhythmic condition plays a pivotal role in the development of LVSD. A low AF burden as well as atrial septal regional fibrosis were identified as predictors of AIC in these patients.

## Profiling hypotension in heart failure: beyond the office reading

Hypotension remains a key concern in the management of HF with reduced ejection fraction (HFrEF), often limiting the optimization of guideline‐directed medical therapy (GDMT). Indeed, hypotension remains the most frequently perceived clinical barrier to implementation of GDMT before creatinine increase and hyperkalaemia.^6^ Contrary to this perception, the prevalence of low systolic BP <90 mmHg was shown to be very low (1.8% of HF patients) in a real‐world cohort.^7^ This discrepancy raises the question of whether conventional office BP measurements truly reflect the burden of hypotension in daily life.

In a recent research letter, Soloveva *et al*.^8^ profiled hypotension in 79 well‐treated HFrEF patients using office, orthostatic, and ambulatory BP monitoring (ABPM). Strikingly, office systolic BP <90 mmHg was observed in only 3.8% of patients, while nearly half experienced postprandial hypotension and 46% had daytime episodes of systolic BP <90 mmHg on ABPM. Importantly, only a quarter of patients were free of any hypotensive episodes. Symptoms such as dizziness and fatigue were common, but did not consistently align with BP drops, suggesting that patient complaints may reflect the underlying HF itself rather than hypotension *per se*.

Clinically, these findings underscore the limitations of relying on office BP alone when titrating HF therapy. ABPM appears critical for uncovering ‘hidden’ hypotension, yet the presence of low BP should not automatically prompt down‐titration. Registry data from more than 42 000 patients show that low systolic BP is less detrimental when patients are on optimized doses of HF medications, emphasizing that low BP alone should not derail medication optimization.^9^ Instead, clinicians should focus on maintaining GDMT, while addressing triggers such as meals, dehydration, or prolonged standing.

## Association of clonal haematopoiesis with incident heart failure and prognosis

Clonal hematopoiesis of indeterminate potential (CHIP) is caused by acquired mutations in haematopoietic stem cells that yield clonal progeny of mutant leucocytes.^10^ Although CHIP carriers typically display normal haematological indices and remain clinically inapparent,^11^ CHIP is strongly linked with age‐related chronic diseases and haematologic malignancies. However, evidence linking CHIP with HF yielded conflicting findings.^12^, ^13^, ^14^ To this purpose, Karakasis and colleagues conducted a systematic review and meta‐analysis to evaluate the association of CHIP with the incidence and clinical outcomes of HF.^15^

The authors evaluated a total of 13 studies based on five cohorts encompassing 57 755 individuals. Regardless of prior history of coronary artery disease, participants with CHIP had significantly greater risk of new‐onset HF compared to the non‐CHIP group (hazard ratio [HR] 1.23, 95% confidence interval [CI] 1.12–1.35, *p* < 0.0001). No significant subgroup differences for incident HF were observed based on LVEF or age. A subgroup analysis of gene‐specific CHIP subtypes revealed significant differences among ASXL1, DNMT3A, TET2, and JAK2 regarding the risk of incident HF (*p* subgroup = 0.03). While ASXL1, TET2, and JAK2 were associated with a significantly increased risk of incident HF, DNMT3A demonstrated a non‐significant effect (HR 1.13, 95% CI 0.99–1.28). However, it should be noted that the studies included in the meta‐analysis utilized different sequencing methodologies, which may influence the detection of these mutations. Interestingly, a total of four cohorts, including 362 participants, also investigated the association of CHIP with the composite outcome of all‐cause mortality and hospitalization for HF in patients with preexisting baseline HF. Participants with CHIP had a significantly higher risk of the composite outcome compared to the non‐CHIP group (HR 1.84, 95% CI 1.25–2.70, *p* = 0.002).

Therapeutic targeting of CHIP is a new area of research with several promising strategies. One approach involves the inhibition of overactive inflammatory pathways related to the presence of CHIP.^16^ While this method does not directly target CHIP, it alleviates the adverse effects caused by inflammation. Another approach attempts to eliminate the mutated blood cells through selective targeting.^17^ This strategy hinges on the accurate identification of cell surface antigens unique to CHIP cells. In conclusion, the study by Karakasis *et al*.^15^ highlights the emerging importance of CHIP for HF risk stratification and its potential role as a biomarker for more personalized approaches.

## Longitudinal blood pressure and cardiovascular outcomes in heart failure

Management of BP in patients with HF remains controversial, with conflicting evidence regarding optimal BP targets across HF phenotypes.^18^, ^19^ Li *et al*.^20^ conducted a large individual patient data meta‐analysis pooling 28 406 participants from eight landmark randomized clinical trials, encompassing the full spectrum of LVEF. Using time‐dependent Cox regression models, the study examined the association between longitudinal systolic and diastolic BP and key outcomes, including cardiovascular death and HF hospitalization.

The analysis revealed a non‐linear, phenotype‐specific relationship between BP and outcomes. In patients with HFrEF, low systolic BP (<120 mmHg) was consistently associated with increased risk of cardiovascular death and HF hospitalization (HR 1.71, 95% CI 1.60–1.82, *p* < 0.001), whereas higher systolic BP (>140 mmHg) was not linked to worse outcomes. Conversely, in HF with mildly reduced or preserved ejection fraction (HFmrEF/HFpEF), both low and high systolic BP were associated with adverse outcomes (HR 1.74, 95% CI 1.47–2.07, *p* < 0.001 and HR 1.77, 95% CI 1.45–2.17, *p* < 0.001; respectively), producing a U‐shaped risk curve. Similar trends were observed for diastolic BP.

These findings underscore the limitations of uniform BP targets in HF patients and highlight LVEF as a key modifier of BP–outcome relationships. For HFrEF, low BP likely reflects advanced disease and impaired cardiac output, whereas in HFmrEF/HFpEF, both hypotension and uncontrolled hypertension contribute to risk. The authors suggest that dynamic, phenotype‐tailored BP management strategies may improve outcomes, but emphasize the need for prospective trials to define optimal BP targets in contemporary HF populations.
